# Tridimensional models and radiographic study of dorsal laminectomy and thoracolumbar hemilaminectomy in dogs

**DOI:** 10.1590/acb382623

**Published:** 2023-08-04

**Authors:** Ricardo Ysaac García Núñez, Katherine Rasia Gonzales Córdova, Yuri Karaccas de Carvalho

**Affiliations:** 1 Universidade Federal do Acre – Graduate Program in Animal Health and Production – Rio Branco (AC), Brazil.; 2 Universidad Nacional San António Abad del Cusco – Cusco, Peru.; 3 Universidade Federal do Acre – Centro de Ciências Biológicas e da Natureza – Rio Branco (AC), Brazil.; 4 Universidade Federal Fluminense – Faculdade de Medicina Veterinária – Departamento de Patologia e Clínica Veterinária – Niterói (RJ), Brazil.

**Keywords:** Spinal Cord Compression, Orthopedic Procedures, Models, Educational, Education, Veterinary

## Abstract

**Purpose:**

To create three-dimensional anatomical models of the thoracic and lumbar portions of the canine spine that reproduce the vertebral surgical approaches of dorsal laminectomy and hemilaminectomy, and to perform the respective radiographic evaluations of each approach.

**Methods:**

In a digital archive of the canine spine, digitally replicate the dorsal laminectomy and hemilaminectomy in the thoracic and lumbar portions and, then, make tridimensional prints of the vertebral models and obtain radiographs in three dorsoventral, ventrodorsal and laterolateral projections.

**Results:**

The anatomical models of the surgical spinal canal accesses of the thoracic and lumbar portions showed great fidelity to the natural bones. The created accesses have the proper shape, location and size, and their radiographic images showed similar radiodensities.

**Conclusions:**

The replicas of the dorsal laminectomy and hemilaminectomy developed in the anatomical models in the thoracic and lumbar portions are able to represent the technical recommendations of the specialized literature, as well as their respective radiographic images, which have certain radiological properties that allow to make a deep radiological study. Therefore, the models are useful for neurosurgical training.

## Introduction

Intervertebral disc extrusion is one of the most common neurological problems encountered in veterinary clinical practice. Previously, numerous descriptions of surgical approaches to address various orthopedic pathologies in canine thoracolumbar intervertebral disc extrusion have been published, like dorsal laminectomy (DL) and hemilaminectomy (HL), to remove herniated disc material^1^.

The tridimensional (3D) printing technique has the power to make the manufacturing process a highly repeatable and consistent process, which results in more effective clinic and better patient outcomes^2^. In addition, this technology allows to improve pre-surgical planning, design custom surgical tools and specific implants, practice and explore various surgical approaches^3^.

The rise of 3D printing in design and manufacturing is a product of the vision and implementation of pioneers who made it a reality, evolving exponentially for its potential and its application in almost every field of manufacturing and customization such as aeronautics, engineering, architecture, pharmacy, and medicine^4^. In this field precisely, educational, and freely accessible materials for spine procedures have been created^5^.

Thus, in recent decades, the application of 3D printing in spine surgery has grown rapidly, demonstrating its effectiveness in minimally invasive approaches. There are many practical uses of this technology within spine surgery. Considering them, anatomical models offer a visual and tactile sense of the specific surgical anatomy for operative planning, patient counseling, and surgical education and training^6^.

## Methods

The research was developed at the Universidade Federal do Acre, Rio Branco (AC), Brazil, and was not submitted to the Ethics Committee on Animal Use, because no live animal was used for experimentation.

The digital file of the spine of a medium-sized canine, which was the raw material for the replication of the anatomical models of spinal canal surgical approaches, is the property of the 3D Educational Technologies Laboratory of the university and part of its digital image collection.

Following the recommendations of all modifications^7^, corrections, replications, and manipulation of the digital files were done using the free-to-use software Autodesk® MeshmixerTM, version 3.5.474 (Autodesk Inc, California, United States of America, 2017). Initially from the original digital file with the .obj extension of the complete canine spine, the thoracic portion of the spine (TPS) and the lumbar portion of the spine (LPS) were obtained, which were saved independently with the same .obj extension.

In each digital file of each spinal portion, the DL and HL were replicated, following the authors’ technical indications in detail^8^^,^ 9. Thus, in each of the replicated accesses, the established measurements and recommendations were maintained exactly.

In the TPS and due to an error in the original file, it was only possible to obtain five vertebrae. The DL was done between the first and second vertebrae, and the HL between the third and fourth, on the left side of the spinous processes.

The LPS has the seven complete vertebrae that characterize the canines, and the surgical approaches were replicated between the second and third vertebrae the DL, and between the fifth and sixth vertebrae the HL on the left side of the dorsal spinous processes.

After obtaining the replicas of the surgical approaches in each anatomical segment, each model was printed with polylactic acid (PLA) filament in a GT Max3D three-dimensional printer model H4 (GT Max3D^©^, Brazil) that uses proprietary software, and the 3D printing technique fused deposition modeling (FDM).

From each anatomical models of spinal canal surgical approaches (AMSCSA) obtained, radiographs were taken from three different radiographic projections, dorsoventral (DV), ventrodorsal (VD) and laterolateral (LL); in an ECORAY brand digital X-ray machine model ULTRA 100 HF (ECORAY^©^, Korea), exposure with 60 kv of radiation intensity and exposure time of 3.2 mAs. RadiAnt DICOM Viewer software (Medixant^©^, Poland) was used to visualize the digital radiographic images.

## Results

The specific morphological structures of each of the AMSCSA are completely visible and distinguishable. Thus, the transverse processes, spinous processes, articular processes, vertebral bodies, and the vertebral foramen of each vertebra are easily observable. The impression parameters are shown in Table 1.

**Table 1:** Impression parameters of the anatomical models of the surgical approaches of the vertebral column.

Impression parameters		Anatomical model	
		Toracic	Lumbar
Impression temperature (°C)		205	205
Layer height (mm)		0,1	0,1
Filling (%)		100	100
Printing speed (mm/s)		50	50
Filament type		PLA	PLA
Amount of filament used	(g)	92* g	283 g
	(m)	31*	94,87
Printing time		28 hours/10 minutes*	85 hours/16 minutes
Model length (cm)		14	24,50
Final cost ($)		10	14

* Taking into consideration that only five vertebrae were printed; PLA: polylactic acid. Source: Authors.

**Figure 1:**
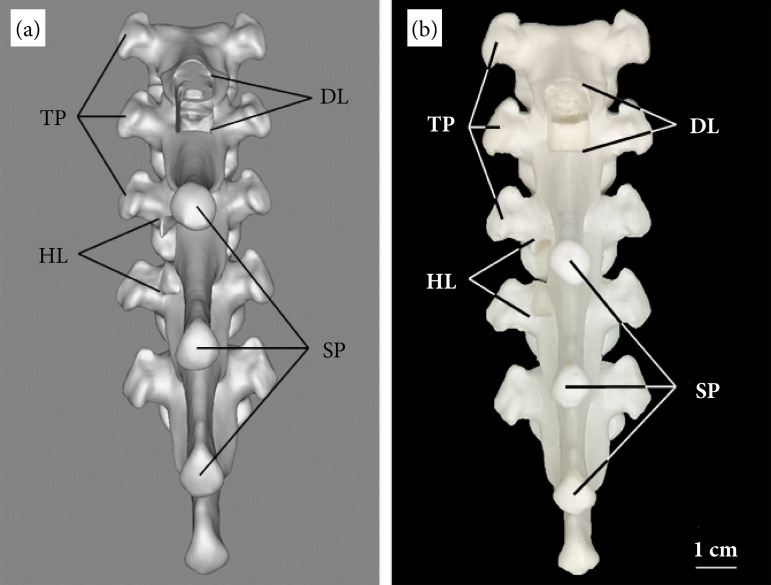
Dorsal views of the tridimensional anatomical model of the thoracic portion of the spine. **(a)** Digital model, **(b)** printed model.

TP: transverse process; SP: spinous process; DL: dorsal laminectomy; HL: hemilaminectomy. Source: Authors.

The anatomical model of the TPS is about 14 cm long, and, according to the literature consulted, the DL was represented as a rectangular opening in the dorsal surface and cranial-caudal projection, with 15 mm in length and 10 mm in width, gaining access to the medullary canal and leaving it exposed for surgical manipulation. As the access recommends, the dorsal laminae of the cranial and caudal vertebrae were removed. In the case of the cranial vertebra, the removed portion of the lamina contained the entire spinous process, the same one that was removed to give the right shape for surgical access. The portion of blade extracted from the caudal vertebra was small and encompassed the entire cranial articular process, but it did not affect the spinous process of this part (Fig. 1).

The HL made on the anatomical model of the TPS was located to the left side of the spinous process and was also rectangular in shape, with a cranial caudal projection of 20 mm and 5 mm in length and width, respectively. The access allows to clearly visualize the spinal canal. By the dorsolateral position of the opening made, the access communicates with the intervertebral foramen.

**Figure 2:**
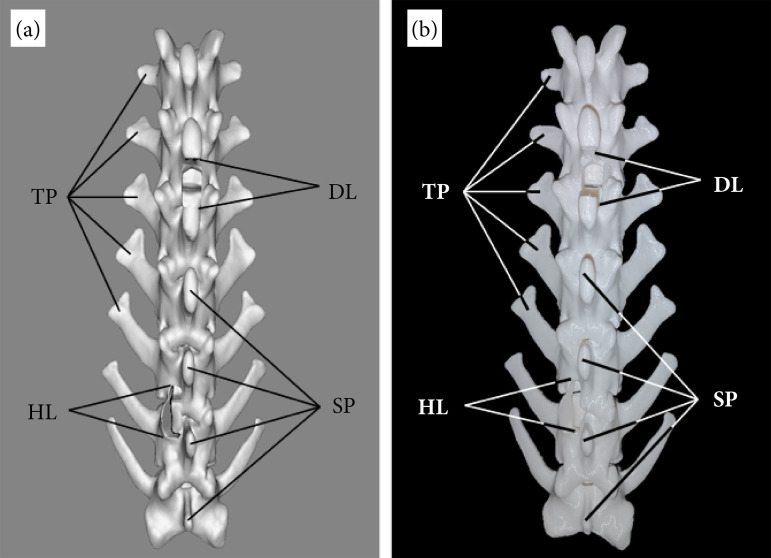
Dorsal views of the tridimensional anatomical model of the lumbar portion of the spine. **(a)** Digital model, **(b)** printed model.

TP: transverse process; SP: spinous process; DL: dorsal laminectomy; HL: hemilaminectomy. Source: Authors.

In the anatomical model of the LPS, the medullary surgical approaches have the following particularities: the DL was replicated dorsally between the L2 and L3 vertebral pieces, was rectangular in shape with a well-defined cranio-caudal projection, 21 mm long, 11 mm wide, and deep enough to access the medullary canal. When removing the dorsal laminae of the lumbar vertebrae, in L2 the removed fragment went with the caudal third of its spinous process, whereas in L3 the extracted fragment had almost half of the cranial portion of its spinous process (Fig. 2).

The HL of the LPS was performed between L5 and L7 on the left side of the spinous processes. It had an enlarged rectangular shape of cranio-caudal projection 25-mm long and 10-mm wide. The access is noted to be clean and well defined with complete visualization of the medullary canal. At L5, the portion of lamina removed encompasses the entire left caudal articular process. On the other hand, at L6, half of the cranial articular process was removed.

The radiographs obtained from the AMSCSA showed the characteristic radiodensities of radiographic studies. In the case of the TPS model, the three standardized projections clearly showed the typical anatomical structures; the spinous processes were clearly differentiated in the LL projection, as were the vertebral bodies. In the DV and VD projections, they clearly showed the small lateral spinous processes.

**Figure 3:**
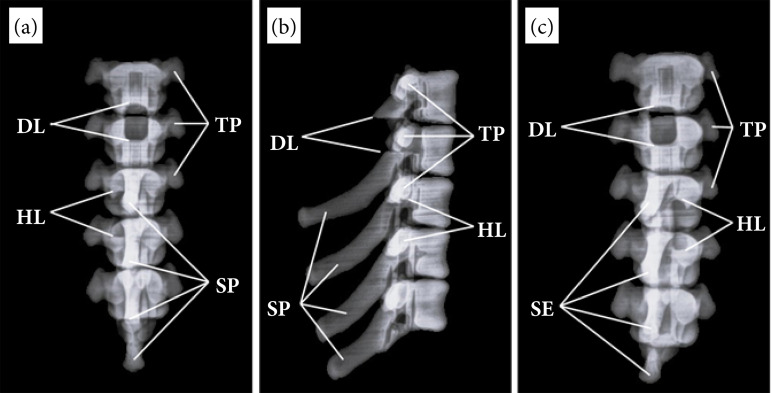
Radiographs of the anatomical model of the thoracic portion of the spine. **(a)** Dorsoventral projection, **(b)** laterolateral projection, **(c)** ventrodorsal projection.

TP: transverse process; SP: spinous process; DL: dorsal laminectomy; HL: hemilaminectomy. Source: Authors.

In the case of the replicated surgical approaches, in the TPS the DL was perfectly visible in all three projections and its described rectangular shape was distinguishable, in the DV and VD projections between the first two vertebrae. In the LL projection, the laminectomy was distinguishable by the complete absence of the spinous process of the first vertebra and the cranial and caudal limits of the access that were appreciated as straight shaped sections (Fig. 3).

The HL done in LPS was on the left side between the third and fourth vertebrae. In the DV projection, the cranial and caudal borders of the replicated access were clearly seen, but the lateral borders were not visible; the left border disappeared due to continuity with the intervertebral foramen and the right lateral border due to the superposition of the respective spinous processes.

**Figure 4:**
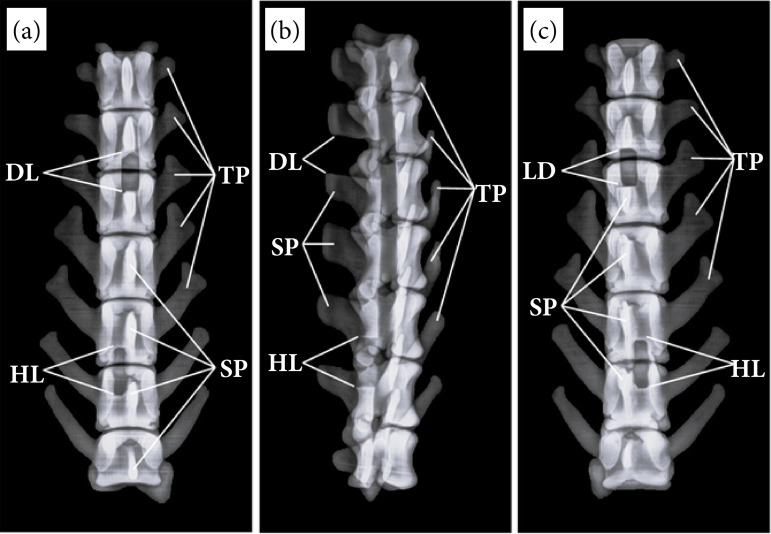
Radiographs of the anatomical model of the lumbar portion of the spine: **(a)** Dorsoventral projection, **(b)** laterolateral projection, **(c)** ventrodorsal projection.

TP: transverse process; SP: spinous process; DL: dorsal laminectomy; HL: hemilaminectomy. Source: Authors.

In the VD projection, the left cranial, caudal, and lateral borders were perfectly noticeable, but the right border did not appear due to its continuity with the intervertebral foramen. In the LL image, the visualization of the surgical access was more difficult; the cranial border was straight and defined; however, the caudal border required a more detailed observation for differentiation (Fig. 4).

The AMSCSA of the LPS in its three radiographic projections clearly showed each of the surgical approaches to the spinal canal, with its characteristic rectangular shape, clean and well-defined borders, well differentiated on radiographic images.

## Discussion

As shown^10^, we have demonstrated the fidelity and accuracy in bone tissue replication by obtaining quality radiographic images of the TPS and LPS that represent the specific anatomical structures in the AMSCSA, which have the enormous potential to become complementary educational tools of surgical anatomy.

Thus, the AMSCSA of the TPS and LPS of this research have the academic value of being important training and teaching tools in the clinical setting, besides being also used in the support of investigations that understand medical images and their diagnostic interpretation according to the manifested by Bücking *et al*.^11^.

In this sense, the creation of these types of specific anatomical spinal models is the first step in a successful surgical procedure, by mimicking the technique and preparing the student or expert surgeon who requires quality prior training, achieving a realistic visual and haptic simulation as manifested^12^.

Optimal skills in successfully performing deep surgical procedures, therefore, require thorough and adequate training aimed at mastering the technique and decreasing the risks to patients in the operating room. Therefore, having the AMSCSA at hand improves surgical preparation and training, generating precision and efficiency of deep procedures, and minimizing the risk for patients^13^.

According to our bibliographical research, we did not find any research that replicates the DL in anatomical models, but there are studies that have developed specific drilling guides for placement of screws in the DL around the laboratory, and instruments that demonstrate their potential to improve and simplify surgical steps with accuracy, less time, and decreased costs, as well as our AMSCSA^14^.

In HL’s case^15^, they developed a patient specific drill guide that also uses 3D printing in order to evaluate its accuracy and safety. Finding no significant differences between two groups of surgeons with different prior experience when placing the screws, they concluded that the specific 3D printed guides have a positive evaluation and, therefore, can be an alternative for training and surgical preparation, which, together with specific replicas of the procedures, further improves the previous preparation.

The high fidelity of the radiographic images obtained from AMSCSA of TPS and LPS and the visualization of similar structures agree with the results obtained by Varallo *et al*.^16^, who fabricated breast models with the same FDM 3D printing technique, but using different types of filament, which showed realistic textures to radiographic exposure in digital mammography and breast CT studies.

The combination of 3D printing and medical imaging studies results in a powerful diagnostic tool and specialized medical training, significantly impacting many fields of medicine, creating anatomical models that help in complicated surgeries and teaching delicate procedures mainly^17^, hence the importance of having radiographic studies of the AMSCSA done and that corroborate the fidelity of the proposed surgical approaches.

However, in the surgical training field, the desired dexterity in surgeons is through experience acquired in the operating room itself. In this sense, the exposure of students to complex cases and pathologies varies greatly among different training programs^18^. Thus, it is necessary to provide the students with tools, such as the AMSCSA of TPS and LPS, for a previous exposure and specific training.

In this context, the use of 3D printing technology in orthopedics becomes a reality. Surgeons can expect an exponential increase in its applications in the coming years. Those are expanding rapidly with increasing benefits for the surgeons themselves and the patients. Its main uses nowadays are in education, orthopedics, surgical planning, the creation of specific guides, and 3D printed implants. Therefore, it is important that all orthopedic surgeons know about this technology^19^.

The AMSCSA of the PTC and LPS created in this research by 3D printing technology are anatomically accurate. It has the potential to aid in the teaching-learning process of residents and support surgical planning in spinal neurosurgery, as noted by McMenamin et al.^20^.

As mentioned^21^, spinal osteotomy is still a risky technique that can lead to injuries and requires previous training. Therefore, AMSCSA of TPS and LPS are innovatively useful tools to expose students to the most common surgical techniques for spinal cord decompression.

Finally, the usefulness of the AMSCSA of TPS and LPS is based on the fact that the most active field in neurosurgery is spinal diseases, as well as the development of countless investigation that discuss the use and application of neurosurgical disease models using 3D printing technology today^22^.

## Conclusion

The DL and HL replicas developed in the AMSCSA of TPS and LPS managed to represent the technical recommendations of the specialized bibliography. They were perfectly visible and distinguishable, as well as their respective radiographic images, which had certain radiological properties that allow to make a deep, detailed and differentiated radiological study. Therefore, the models are useful for neurosurgical training that can complement the preparation and specialized training.
